# Predictors of Mortality in Patients with Interstitial Lung Disease-Associated Pulmonary Hypertension

**DOI:** 10.3390/jcm9123828

**Published:** 2020-11-26

**Authors:** Esam H. Alhamad, Joseph G. Cal, Nuha N. Alrajhi, Waleed M. Alharbi

**Affiliations:** 1 Department of Medicine, Division of Pulmonary Medicine, College of Medicine, King Saud University, Riyadh 11461, Saudi Arabia; josephcal_md@yahoo.com (J.G.C.); nuha-alrajhi@hotmail.com (N.N.A.); 2 Department of Cardiac Science, College of Medicine, King Saud University, Riyadh 11461, Saudi Arabia; waleed.alharbi@me.com

**Keywords:** pulmonary hypertension, idiopathic pulmonary fibrosis, interstitial lung disease, six-minute walk test, survival, pulmonary vascular resistance

## Abstract

Background: Pulmonary hypertension (PH) is a well-established complication in interstitial lung disease (ILD) patients. The aim of this study is to investigate the physiological and hemodynamic parameters that predict mortality in patients with ILD-PH. Methods: Consecutive ILD patients who underwent right heart catheterization (*n* = 340) were included. The information analyzed included demographics and physiological and hemodynamic parameters. Cox regression models were used to identify independent predictors of survival. Results: In total, 96 patients had PH and an additional 56 patients had severe PH. The overall survival of idiopathic pulmonary fibrosis (IPF) patients with PH was significantly worse than the survival of patients with other types of ILD with PH (*p* < 0.0001 by log-rank analysis). Patients with a reduced diffusing capacity of the lung for carbon monoxide (DLco) (<35% predicted), six-minute walk test final oxygen saturation by pulse oximetry (SpO_2_) < 88% and pulmonary vascular resistance ≥4.5 Wood units in the ILD-PH cohort had significantly worse survival. IPF diagnosis, forced vital capacity, DLco, systolic pulmonary artery pressure and cardiac index were identified as independent predictors of survival among the ILD-PH cohort. Conclusions: Patients with ILD-PH have poor prognosis. Physiological and hemodynamic parameters were important factors independently associated with outcome.

## 1. Introduction

Interstitial lung disease (ILD) comprises a group of disorders that affect the lung parenchyma with varying degrees of pulmonary fibrosis. Idiopathic interstitial pneumonia, connective tissue disease (CTD)-associated ILD, sarcoidosis and chronic hypersensitivity pneumonitis represent the vast majority of ILD cases seen in the ILD clinic. When evaluating ILD patients, clinicians commonly face significant challenges in identifying the cause of progressive worsening of dyspnea, whether it is related to a progressive ILD course or is a consequence of comorbidity that worsened the natural course. For example, pulmonary hypertension (PH), defined as a mean pulmonary artery pressure (mPAP) ≥ 25 mmHg, is one of the most common comorbidities encountered in ILD patients and leads to worsening of dyspnea, decreased functional capacity, increased need for oxygen supplementation and ultimately reduced survival [1,2,3,4,5,6].

Recently, the Sixth World Symposium on Pulmonary Hypertension task force revised the definition of PH for chronic lung disease (Group 3) to mPAP > 20 mmHg with pulmonary vascular resistance (PVR) ≥ 3 Wood units (WU) [7]. However, the clinical significance and prognostic impact of changing the definition in Group 3 PH is unclear.

In this context, we reviewed a series of consecutive ILD patients evaluated in one center who underwent right heart catheterization (RHC) while applying the new PH definition to determine the physiological and hemodynamic parameters that predict survival in ILD associated PH patients. ILD patients without PH confirmed by RHC evaluated during the same time period were chosen as a control for comparison purposes.

## 2. Methods

The present study is a retrospective review of the ongoing prospective ILD and PH registry at the ILD and PH Centre at King Saud University Medical City. Consecutive ILD patients diagnosed with PH based on RHC between 1 February 2008 and 31 October 2019 were included. RHC was performed within 7 days of establishing an ILD diagnosis or during ILD follow-up when PH was suspected. RHC parameters were obtained from ILD patients in a stable condition. All PH cases presented in this study are incident cases. The study was conducted in accordance with the Declaration of Helsinki (as revised in 2013). This study was approved by the Institutional Research Board at the College of Medicine, King Saud University, Riyadh, Saudi Arabia (approval number E-20-4608). The need to obtain written informed consent was waived because of the retrospective nature of the current study.

The demographics collected included age, sex and smoking history. Physiological studies included forced vital capacity (FVC), forced expiratory volume in one second (FEV_1_), FEV_1_/FVC ratio, and diffusion capacity of the lung for carbon monoxide (DLco) [8,9,10]. In addition, 6 min walk test (6MWT) parameters, including initial and final oxygen saturation by pulse oximetry (SpO_2_) and 6 min walk distance (6MWD), were collected [11]. Diagnosis of idiopathic pulmonary fibrosis (IPF) and other ILD subtypes was established by a multidisciplinary approach after a thorough analysis of clinical radiological, histopathological (when available) and serological test results according to established guidelines [12,13,14,15,16,17,18,19,20,21,22]. Only sarcoidosis cases with advanced fibrocystic disease (i.e., radiographic Scadding stage 4) were included in the current study.

Patients were categorized as without PH (defined as the mPAP < 21 mmHg, or mPAP 21–24 mmHg with PVR < 3 WU), with PH (defined as mPAP 21–24 mmHg with PVR ≥ 3 WU, or mPAP 25–34 mmHg) and with severe PH (defined as mPAP ≥ 35 mmHg or mPAP ≥ 25 mmHg with low cardiac index (<2.0 L/min/m^2^)) as previously described [7]. PVR was calculated as the difference between mPAP and pulmonary capillary wedge pressure (PCWP) divided by the cardiac output. Patients with post-capillary PH (pulmonary capillary wedge pressure (PCWP) > 15 mmHg and PVR < 3 WU, *n* = 26) were excluded.

### Statistical Analysis

Data are presented as the means ± standard deviations or numbers (percentages), where appropriate. Between-group differences were compared using *t*-test, one-way analysis of variance (ANOVA), Chi-square test or Fisher’s exact test, as appropriate. Kaplan–Meier survival curves and log-rank tests were used to investigate the time from the initial diagnosis of PH to death, transplant, loss to follow-up, or end of the study period (i.e., follow-up duration). Survival status was determined by contacting the patient or was retrieved from medical records. Survival time was censored on 31 May 2020, at the time the patient underwent lung transplant, if they were lost to follow-up, or at the date of the last visit. Cox proportional hazard regression models were used to calculate hazard ratios (HR) on all-cause mortality. Univariate parameters with a *p*-value < 0.05 were considered for inclusion in stepwise forward multivariate Cox proportional hazards model to identify the independent predictors of mortality among the PH patients. Two-sided *p* values < 0.05 and 95% confidence intervals were used to report the statistical significance and precision of our results, respectively. The SPSS (Statistical Package for the Social Sciences) version 18 software package (SPSS Inc., Chicago, IL, USA) was used for all analyses.

## 3. Results

A total of 340 patients underwent RHC, of which 96 patients had PH and 56 had severe PH. On average, the follow-up of our PH patients was 41 months, with a maximum follow-up of 11 years. Among the PH group (*n* = 96), 21 patient met the new criteria for PH (defined as mPAP 21–24 mmHg with PVR ≥ 3 WU). In the CTD-ILD patients, 72 patients had the usual interstitial pneumonia pattern (without PH, *n* = 38; with PH, *n* = 23; severe PH, *n* = 11), 53 patients had a nonspecific interstitial pneumonia pattern (without PH *n* = 30; with PH, *n* = 14; severe PH, *n* = 9) and 4 patients had lymphocytic interstitial pneumonia (without PH, *n* = 2; with PH, *n* = 2).

The baseline demographic characteristics of the ILD patients in the without PH, with PH and severe PH groups are summarized in Table 1.

Compared to ILD patients without PH, marked physiological impairments in pulmonary function tests (PFTs) and 6MWT parameters were noted in the PH and severe PH groups (Table 2).

Regarding the use of PH-specific therapy, no significant difference was noted between the group with PH and that with severe PH (Table 2). However, oxygen supplementation was prescribed significantly more in patients with PH and severe PH than in those without PH (*p* < 0.0001) (Table 2). A comparison of hemodynamic parameters between the three groups is shown in Table 3.

### Survival Analysis of the ILD Cohort

**Figure 1:**
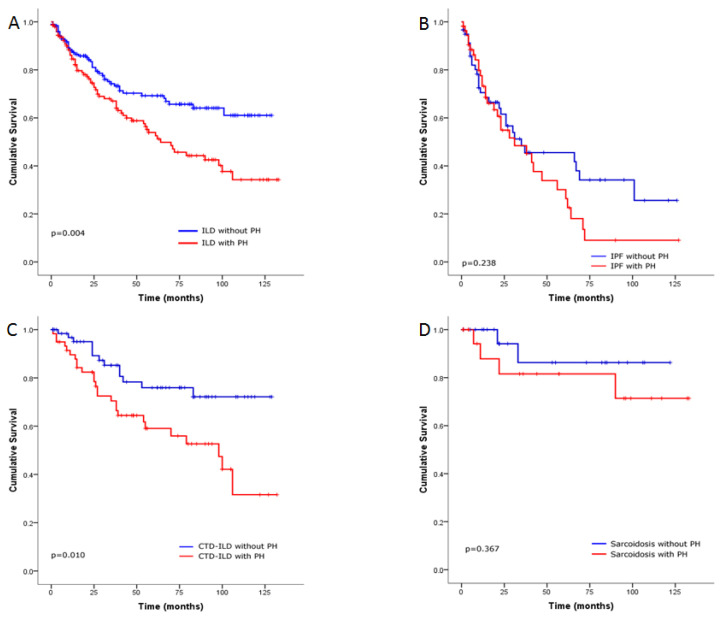
Kaplan–Meier survival estimates for the relationship with (**A**) interstitial lung disease (ILD) patients with and without pulmonary hypertension (PH), (**B**) idiopathic pulmonary fibrosis (IPF) patients with and without PH, (**C**) connective tissue disease (CTD) associated ILD patients with and without PH and (**D**) sarcoidosis patients with and without PH.

In total, 111 patients died (without PH, *n* = 47; with PH, *n* = 33; severe PH, *n* = 31), and two underwent transplantation. The estimated survival probabilities at 1, 3 and 5 years were 84%, 71%, and 66%, respectively, in the ILD without PH group; and 75%, 59%, and 47%, respectively, in the PH group (with PH and severe PH) (*p* = 0.004 by log-rank analysis; Figure 1A). When we examined ILD patients according to the underlying disease, the estimated survival probabilities at 1, 3, and 5 years among IPF patients without PH were 62%, 46%, and 34%, respectively, and 56%, 36%, and 15%, respectively, in the IPF patients with PH (*p* = 0.238 by log-rank analysis; Figure 1B); 95%, 78%, and 76%, respectively, in the CTD-ILD without PH and 82%, 64%, and 56%, respectively, in the CTD-ILD with PH (*p* = 0.010 by log-rank analysis; Figure 1C); 94%, 87%, and 87%, respectively, in the sarcoidosis without PH and 83%, 83%, and 83%, respectively, in the sarcoidosis patients with PH (*p* = 0.367 by log-rank analysis; Figure 1D).

**Figure 2:**
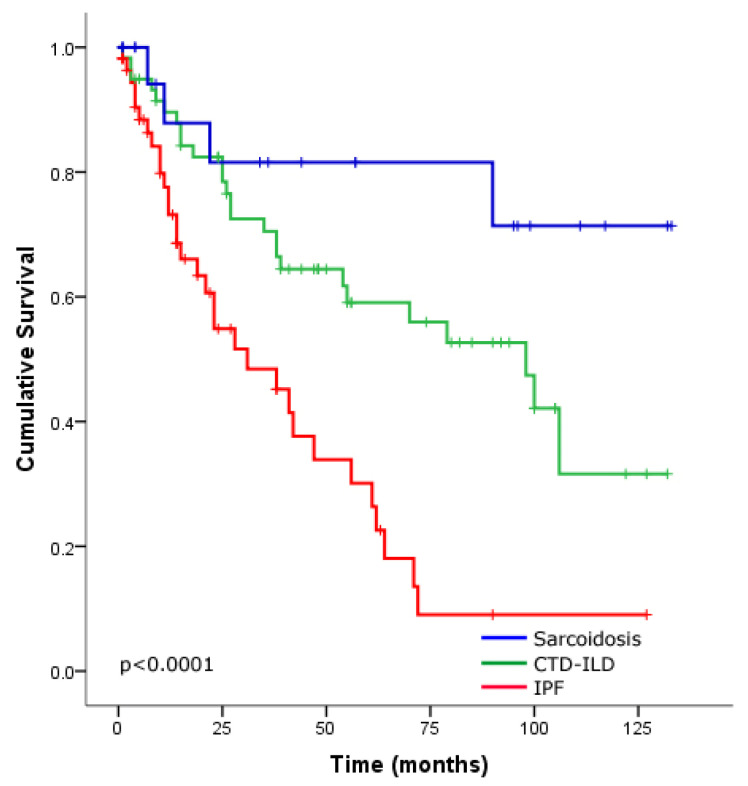
Kaplan–Meier survival estimates for interstitial lung disease (ILD) patients with pulmonary hypertension according to the underlying disease idiopathic pulmonary fibrosis (IPF) (red line), connective tissue disease (CTD) associated ILD (green line) and sarcoidosis (blue line).

The overall survival among PH patients (with PH and severe PH) was significantly different between IPF, CTD-ILD and sarcoidosis (*p* < 0.0001 by log-rank analysis; Figure 2). The survival in IPF-PH patients was significantly worse than the survival of patients with CTD-ILD and sarcoidosis with PH (*p* < 0.0001, *p* < 0.0001, respectively, by log-rank analysis; Figure 2). While sarcoidosis patients with PH tended to survive longer than those with CTD-ILD patients with PH, this difference was not significant (*p* = 0.064 by log-rank analysis) (Figure 2).

**Figure 3:**
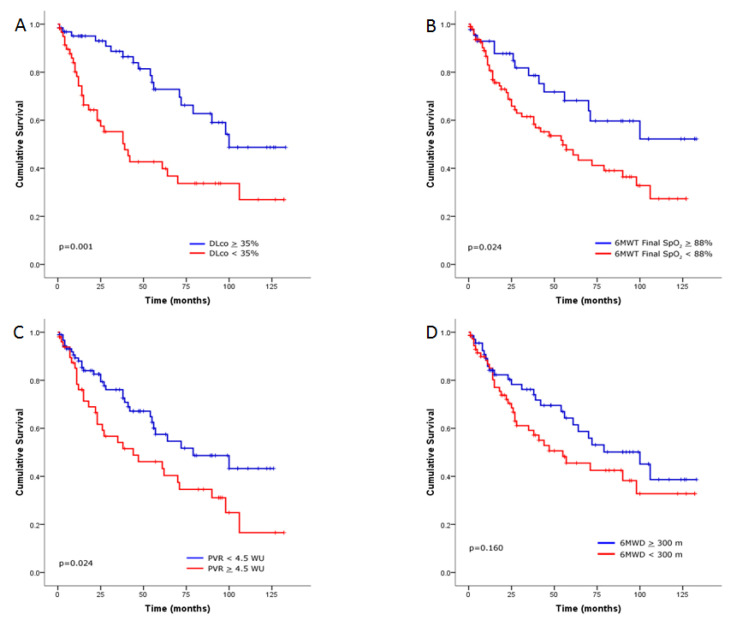
Kaplan–Meier survival estimates for the relationship with (**A**) diffusion capacity of the lung for carbon monoxide (DLco) at a threshold of 35% predicted, (**B**) six-minute walk test (6MWT) final oxygen saturation by pulse oximetry (SpO_2_) at a threshold of 88%, (**C**) pulmonary vascular resistance (PVR) ≥ 4.5 Wood units (WU) and (**D**) 6 min walk distance (6MWD) at a threshold of 300 m in interstitial lung disease patients with pulmonary hypertension.

Survival in the entire ILD-PH cohort (with PH and severe PH) revealed that physiological and hemodynamic parameters were predictors of worse outcome, including DLco < 35% predicted (*p* = 0.001 by log-rank analysis; Figure 3A), 6MWT final SpO_2_ < 88% (*p* = 0.024 by log-rank analysis; Figure 3B) and PVR ≥ 4.5 WU (*p* = 0.024 by log-rank analysis; Figure 3C). However, 6MWD < 300 m was not associated with a worse outcome (*p* = 0.160 by log-rank analysis; Figure 3D).

In the univariate Cox regression analysis, baseline variables significantly predicting outcome among ILD-PH patients (with PH and severe PH) were age, male sex, smoking history, body mass index (BMI), IPF diagnosis, sarcoidosis diagnosis, % predicted FVC, % predicted DLco, 6MWT final SpO_2_ < 88%, systolic PAP, PCWP, PVR, cardiac index and use of immunomodulator therapy and PH-specific therapy. However, in the multivariable analysis, IPF diagnosis, % predicted FVC, % predicted DLco, systolic PAP and cardiac index were independent predictors of survival (Table 4).

In the severe PH group, a univariate analysis revealed that IPF diagnosis, sarcoidosis diagnosis, the percent of predicted DLco, PCWP, cardiac index and use of immunomodulator and PH-specific therapy were significantly associated with survival. In the multivariable analysis, only IPF diagnosis and the percent of predicted DLco remained significantly associated with survival (Table 5).

## 4. Discussion

The present study describes a large cohort of ILD-PH patients with variable degrees of parenchymal fibrosis. We show that 28% of ILD patients had PH and 16% of patients fulfilled the definition of severe PH.

PH due to IPF is well established and is associated with significant morbidity and reduced survival [2,4,23,24]. Estimates suggest that the prevalence of PH among IPF patients ranges between 30% and 50% [25]. In the present study, the incidence of PH among IPF patients was 48.6%, and of these patients, 19% had severe PH. A previous report in IPF patients awaiting lung transplantation showed that severe PH (defined as a mPAP > 40 mmHg) was noted in one in every 10 patients [26]. However, when clinicians encounter severe PH in IPF patients with a significant history of tobacco smoking, careful assessment is needed to consider the possibility of underlying combined pulmonary fibrosis and emphysema (CPFE). Cottin et al. [27] reported that nearly 70% of patients with CPFE have severe PH. In the present study, only 9% of the PH cases fulfil the proposed criteria for CPFE, as previously described [27]. As such, whether the severe IPF-PH cases noted in our study are the result of selection bias towards the most severe cases being referred to our center or whether it reflects a distinct IPF phenotype in this region is not clear. Nonetheless, the observed three-year survival rate of patients in our IPF cohort with PH was 36%, which is similar to the rates in previous studies ranging between 16% and 34% depending on the studied population [1,2,28]. Notably, we found no significant difference in survival between IPF patients with or without PH. The potential explanation is that our IPF patients without PH have an advanced lung fibrosis (mean FVC: 59%, and mean 6MWD: 312 m). In addition, 31.5% of our IPF patients without PH have reduced cardiac index < 2.5 L/min/m^2^, implying that cardiac involvement does occur in IPF patients even in the absence of PH [29]. In agreement with our observation, D’Andrea et al. [30] performed 2D strain echocardiography of the right ventricular (RV) septal and lateral walls among 52 IPF patients and found that impaired RV diastolic and systolic myocardial function were present even in the absence of PH. In another study, right ventricle:left ventricle diameter ratio and RV dysfunction measured by echocardiography predicted adverse outcomes independent of the presence of IPF-PH or the level of the PVR [31]. As such, our findings along with cited studies show that cardiac complications manifested by right-sided heart dysfunction are an important marker of outcome among IPF patients with or without PH. Another important observation noted in our ILD-PH cohorts is that IPF was independently associated with a 2.5-fold increased risk of mortality. Thus, the consensus recommendation is emphasized by the International Society of Heart and Lung Transplant guidelines on candidate selection for transplant to list IPF patients when PH is diagnosed [32].

PH secondary to CTD is a well-recognized complication that is classified under Group 1 PH [33]. PH data from Western countries reveal that systemic sclerosis (SSc) followed by systemic lupus erythematosus (SLE) and mixed CTD represent the vast majority of CTD subtypes, while primary Sjogren’s syndrome (pSS) is rarely reported [34,35]. However, PH studies from Chinese cohorts show that SLE followed by SSc and pSS were the main underlying CTD subtypes [36,37,38]. In the cited studies, the prevalence of pSS-PH (Group 1 PH) ranges between 11% and 16% implying that racial, genetic and environmental factors may have contributed to such differences [36,37,38]. Data on CTD-ILD associated with PH are mainly from SSc patients. Several studies have shown that PH in SSc-ILD is far worse than in SSc-PH without ILD [28,39,40]. In the present study, we show that the presence of PH in CTD-ILD is significantly associated with decreased survival when compared to CTD-ILD patients without PH. Importantly, our PH cohort with CTD-ILD consists primarily of patients with pSS and undifferentiated CTD (UCTD) which contributes to the limited data available amongst these patients, for whom we found the incidence of PH in pSS-ILD was 33.8%. Notably, in the severe PH group, 12.5% of the patients had underlying pSS, highlighting the importance of identifying patients with pSS-ILD when severe PH is encountered. Importantly, the severity of the hemodynamic parameters noted in our pSS-ILD patients (Group 3 PH) is in agreement with other studies [38,41,42] of pSS-PH patients (Group 1 PH), implying that regardless of the underlying PH group classifications, pSS patients are more prone to develop severe PH. Suggested mechanisms that have been implicated in the pathogenesis of PH in pSS patients include vasculopathy, B cell activation, autoimmunity and others [41]. Nonetheless, future studies are needed to understand the mechanism of pSS that leads to severe PH, particularly in ILD patients.

The development of PH in patients with UCTD-associated ILD is another important observation noted in our study. Despite the follow-up in our ILD clinic over an average of 4 years, none of the UCTD patients developed any of the definite CTDs, which is consistent with the natural history of UCTD in which the majority of such cases will remain undifferentiated [43]. Recently, a new term has been proposed in ILD patients with autoimmune manifestations not fulfilling the classification criteria of a given CTD: interstitial pneumonia with autoimmune features (IPAF) [44]. Whether our UCTD patients are distinct from IPAF patients or they represent the same autoimmune disorder entity is beyond the scope of the current study. The prevalence and incidence of PH in UCTD-ILD patients are unknown. In our cohort, the incidence of PH was 27.1%. Furthermore, UCTD patients represent 14.2% of the cases with severe ILD-PH. However, because data on PH in UCTD-ILD patients are limited, future studies in another population are needed to understand the mechanisms leading to severe PH in these patients.

PH secondary to sarcoidosis is a well-recognized complication. In the present study, the incidence of PH among sarcoid patients was 45%, of which 23% had severe PH. The observed 3-year survival rates in the PH-sarcoidosis patients was 83%, which is similar to previous reports describing the outcome among PH-sarcoidosis patients [5,45]. As such, our data imply that despite the parenchymal fibrosis and the severity of hemodynamic parameters observed in sarcoidosis patients, they have a more favorable prognosis than IPF and CTD-ILD patients with PH.

In our ILD-PH cohort, we show that a PVR value ≥ 4.5 WU was significantly associated with a high mortality risk. Although based on univariate analysis, PVR was significantly associated with survival, yet in the multivariate analysis, it failed to emerge as an independent predictor of mortality. This finding is in agreement with previous studies of a cohort of patients with PH due to chronic lung disease [1,46]. Other hemodynamic parameters noted to be independently associated with ILD-PH survival among our cohort were systolic PAP and cardiac index. The association between low cardiac index and increased mortality in ILD-PH patients is consistent with other studies of patients with PH due chronic lung disease [27,46,47], thus emphasizing the importance of early recognition of cardiac involvement in ILD patients. Furthermore, the association between PAP and increased mortality among our ILD-PH cohort compliment the findings of others [4,23,24] which further highlights the importance of hemodynamic parameters that can serve as prognostic markers in ILD-PH patients.

Amongst the physiological variables that emerged as independent predictors of survival, we found the percent of predicted FVC and DLco were important markers of mortality in ILD-PH patients. Importantly, the significant association between the reduced DLco (<35% predicted) and increased mortality observed in our ILD-PH patients is in line with other PH studies and emphasizes the importance of diffusing capacity as a useful physiological parameter for identifying the highest risk of mortality in both ILD and non-ILD patients [5,27,46,48,49].

In the present analysis, nearly 60% of the PH cohort received PH-specific therapy. Interestingly, the association between PH-specific therapy and improved survival was maintained in the univariate analysis even after the exclusion of sarcoidosis patients (HR 0.506, 95% confidence interval 0.298–0.857; *p* = 0.011), implying that PH patients with CTD-ILD may have a favorable response when PH-specific therapy was applied. Nonetheless, our results need to be interpreted with caution, and future studies are needed to examine the response to PH-specific therapy among PH patients with CTD-ILD.

The present study had several strengths and limitations. The strengths include enrolling a large consecutive cohort of ILD patients with and without PH confirmed by RHC in one center. Moreover, the majority of ILD patients underwent RHC within 7 days of establishing an ILD diagnosis. Lastly, the management decision including the use of PH-specific therapy for each patient in our center was discussed in a multidisciplinary meeting after obtaining all the necessary information. Limitations include that all patients in the present study were Saudis; thus, our results, in particular, pSS and UCTD as the two major causes of PH in patients with CTD with ILD, may not be extrapolated to other populations. Second, the retrospective review of the database from one center may introduce data bias, although data were acquired prospectively. Furthermore, independent predictors of mortality could not be determined for the chronic hypersensitivity pneumonitis and idiopathic nonspecific interstitial pneumonia, due to the small sample size and low number of deaths. Finally, our center is highly specialized in the diagnosis and management of various ILDs; thus, selection bias may have occurred due to the most severe cases being referred to our center, which may lead to the overestimation of the incidence and mortality of ILD patients with PH.

## 5. Conclusions

This study describes the PH outcomes in diverse ILD patients with variable degrees of hemodynamic derangements, which highlights a number of important issues pertaining to this serious complication. The overall survival of patients with IPF-PH was significantly worse than the survival of patients with other types of ILD with PH. Physiological (the percent of predicted FVC and DLco) and hemodynamic (systolic PAP and cardiac index) parameters were important factors independently associated with the outcome among ILD-PH patients. Unfortunately, the majority of ILD-PH patients are routinely excluded from clinical trials of PH-specific therapy. Therefore, future studies are highly needed to address the devastating complications of ILD-PH, particularly amongst those with severe PH.

## Figures and Tables

**Table 1:** Demographic characteristics of the study cohort.

Variable	Without PH	With PH	Severe PH	*p*-Value
	(*n* = 188)	(*n* = 96)	(*n* = 56)	
Age	55.8 ± 15.6	60.2 ± 14.3	59.3 ± 14.3	0.042
Male sex	93 (49.5)	36 (37.5)	25 (44.6)	0.158
Ever smoker	46 (24.4)	17 (17.7)	17 (30.3)	0.187
Follow-up duration, months	42.7 ± 38.5	40.8 ± 37.8	41.7 ± 37.5	0.921
BMI, kg/m^2^	28.9 ± 6.3	30.1 ± 6.6	30.4 ± 8.4	0.202
**Underlying disease**				
IPF	59 (31.3)	34 (35.4)	22 (39.2)	0.508
CTD-ILD	70 (37.2)	39 (40.6)	20 (35.7)	0.798
Rheumatoid arthritis	13 (6.9)	4 (4.1)	1 (1.7)	0.272
SLE	5 (2.6)	3 (3.1)	1 (1.7)	0.884
Systemic sclerosis	8 (4.2)	9 (9.3)	0	0.030
Primary Sjogren’s syndrome	10 (5.3)	13 (13.5)	7 (12.5)	0.039
Polymyositis	3 (1.5)	0	1 (1.7)	0.448
MCTD	11 (5.8)	2 (2.0)	2 (3.5)	0.324
UCTD	20 (10.6)	8 (8.3)	8 (14.2)	0.516
Sarcoidosis	26 (13.8)	11 (11.4)	11 (19.6)	0.371
Chronic Hypersensitivity Pneumonitis	14 (7.4)	5 (5.2)	1 (1.7)	0.272
Idiopathic NSIP	4 (2.1)	4 (4.1)	2 (3.5)	0.601
Others				
Organizing Pneumonia	4 (2.1)	2 (2.0)	0	0.547
Unclassifiable fibrosis	6 (3.1)	0	0	0.085
RBILD	3 (1.5)	1 (1.0)	0	0.617
DIP	2 (1.0)	0	0	0.443

Data are presented as the means ± standard deviations or numbers (percentages). PH: pulmonary hypertension; BMI: body mass index; IPF: idiopathic pulmonary fibrosis; CTD: connective tissue disease; ILD: interstitial lung disease; SLE: systemic lupus erythematosus; MCMCTD: mixed connective tissue disease; UCTD: undifferentiated connective tissue disease; NSIP: nonspecific interstitial pneumonia; RBILD: respiratory bronchiolitis interstitial lung disease; DIP: desquamative interstitial pneumonia.

**Table 2:** Clinical characteristics of the study cohort.

Variable	Without PH	With PH	Severe PH	*p*-Value
	(*n* = 188)	(*n* = 96)	(*n* = 56)	
**Pulmonary function test**				
FVC, % predicted	62.3 ± 19.1	54.7 ± 18.1 ^ǂ^	52.9 ± 17.7	<0.0001
FEV_1_, % predicted	67.9 ± 19.3	60.7 ± 19.1 ^ǂ^	59.3 ± 19.1	0.001
FEV_1_/FVC, ratio	88.0 ± 9.3	89.4 ± 8.2 ^ǂ^	88.9 ± 8.4	0.449
DL_CO_, % predicted	45.3 ± 20.7 ^γ^	37.9 ± 17.1 ^κ^	33.3 ± 19.4 ^λ^	<0.0001
**Six-minute walk test**	*n* = 184	*n* = 91	*n* = 54	
Initial Borg score	1.0 ± 1.4	0.9 ± 1.4	0.9 ± 0.9	0.984
Final Borg score	3.4 ± 2.3	3.9 ± 2.4	3.9 ± 2.2	0.153
Initial SpO_2_, %	95.7 ± 2.7	94.8 ± 2.7	94.2 ± 3.8	0.001
Final SpO_2_, %	86.7 ± 7.0	82.9 ± 7.6	82.3 ± 8.0	<0.0001
Distance, meters	343.6 ± 113.6	307.8 ± 103.4	251.0 ± 122.0	<0.0001
**Treatment**				
PDE-5i	-	39 (40.6)	20 (35.7)	0.549
ERA	-	3 (3.1)	3 (5.3)	0.670
Prostanoids	-	0	1 (1.7)	0.368
Combination therapy				
PDE-5i + ERA	-	10 (10.4)	7 (12.5)	0.694
PDE-5i + Prostanoids	-	2 (2.0)	1 (1.7)	1.000
PDE-5i + ERA + Prostanoids	-	3 (3.1)	2 (3.5)	1.000
Antifibrotic therapy	31 (16.4)	16 (16.6)	8 (14.2)	0.909
Immuomodulator therapy	44 (23.4)	31 (32.2)	18 (32.1)	0.245
Oxygen supplementation	106 (56.3)	75 (78.1)	44 (78.5)	<0.0001

Data are presented as the means ± standard deviations or numbers (percentages). FVC: forced vital capacity; FEV_1_: forced expiratory volume in one second; TLC: total lung capacity; DLco: diffusion capacity of the lung for carbon monoxide; SpO_2_: oxygen saturation by pulse oximetry; PDE-5i: phosphodiesterase 5 inhibitor; ERA: endothelin receptor antagonist. ^ǂ^ *n* = 94; ^γ^ *n* = 176; ^κ^ *n* = 80; ^λ^ *n* = 46.

**Table 3:** Hemodynamic parameters of the study cohort.

Variable	Without PH	With PH	Severe PH	*p*-Value
	(*n* = 188)	(*n* = 96)	(*n* = 56)	
RAP, mmHg	4.5 ± 3.0	5.7 ± 3.2	8.5 ± 4.2	<0.0001
sPAP, mmHg	29.3 ± 6.0	41.1 ± 5.7	60.7 ± 14.9	<0.0001
dPAP, mmHg	11.2 ± 3.9	17.2 ± 5.1	25.6 ± 6.6	<0.0001
mPAP, mmHg	18.6 ± 3.5	27.0 ± 3.2	40.0 ± 7.5	<0.0001
PCWP, mmHg	8.5 ± 3.5	9.9 ± 3.5	14.4 ± 7.7	<0.0001
PVR, Wood units	2.1 ± 0.8	3.6 ± 1.2	6.0 ± 3.4	<0.0001
CO, L/min	5.0 ± 1.3	4.8 ± 1.0	4.7 ± 1.8	0.193
CI, L/min/m^2^	2.9 ± 0.6	2.8 ± 0.5	2.7 ± 0.9	0.260

Data are presented as the means ± standard deviations. RAP: right atrial pressure; sPAP: systolic pulmonary artery pressure; dPAP: diastolic pulmonary artery pressure; mPAP: mean pulmonary artery pressure; PCWP: pulmonary capillary wedge pressure; PVR: pulmonary vascular resistance; CO: cardiac output; CI: cardiac index.

**Table 4:** Cox proportional hazards regression analysis showing predictors of mortality in patients with pulmonary hypertension (*n* = 152).

Variable	Univariate Predictors		Multivariable Predictors	
	HR (95% CI)	*p*-Value	HR (95% CI)	*p*-Value
Age	1.027 (1.008–1.046)	0.006		
Male sex	2.426 (1.465–4.016)	0.001	1.931 (0.934–3.992)	0.076
Ever smoker	2.235 (1.291–3.870)	0.004		
BMI, kg/m^2^	0.956 (0.919–0.995)	0.026		
IPF diagnosis	3.292 (1.982–5.468)	<0.0001	2.579 (1.293–5.147)	0.007
CTD-ILD diagnosis	0.751 (0.455–1.240)	0.263		
Sarcoidosis diagnosis	0.320 (0.116–0.885)	0.028		
FVC, % predicted	0.971 (0.955–0.988)	0.001	0.972 (0.949–0.995)	0.016
DL_CO_, % predicted	0.970 (0.954–0.986)	<0.0001	0.978 (0.961–0.995)	0.013
6MWD < 300 m	1.439 (0.862–2.403)	0.164		
6MWT final SpO_2_ < 88%	1.969 (1.081–3.589)	0.027		
mPAP, mmHg	1.023 (0.993–1.054)	0.134		
RAP, mmHg	1.042 (0.977–1.112)	0.210		
sPAP, mmHg	1.017 (1.000–1.035)	0.048	1.023 (1.002–1.045)	0.034
dPAP, mmHg	1.031 (0.994–1.069)	0.107		
PCWP, mmHg	1.045 (1.002–1.091)	0.042		
PVR, Wood units	1.109 (1.021–1.206)	0.014		
CI, L/min/m^2^	0.551 (0.373–0.815)	0.003	0.639 (0.421–0.971)	0.036
Antifibrotic therapy	1.764 (0.988–3.150)	0.055		
Immunomodulator therapy	0.574 (0.337–0.979)	0.041		
PH-specific therapy	0.473 (0.286–0.783)	0.004		
Oxygen supplementation	1.721 (0.898–3.299)	0.102		

HR: hazard ratio; 95% CI: 95% confidence interval. BMI: body mass index; IPF; idiopathic pulmonary fibrosis; CTD: connective tissue disease; ILD: interstitial lung disease; FVC: forced vital capacity; DLco: diffusion capacity of the lung for carbon monoxide; 6MWD: six-minute walk distance; 6MWT: six-minute walk test; SpO_2_: oxygen saturation by pulse oximetry; mPAP: mean pulmonary artery pressure; RAP: right atrial pressure; sPAP: systolic pulmonary artery pressure; dPAP: diastolic pulmonary artery pressure; PCWP: pulmonary capillary wedge pressure; PVR: pulmonary vascular resistance; CI: cardiac index; PH: pulmonary hypertension.

**Table 5:** Cox proportional hazards regression analysis showing predictors of mortality in patients with severe pulmonary hypertension (*n* = 56).

Variable	Univariate Predictors		Multivariable Predictors	
	HR (95% CI)	*p*-Value	HR (95% CI)	*p*-Value
Age	1.020 (0.995–1.046)	0.110		
Male sex	1.787 (0.876–3.646)	0.110		
Ever smoker	1.867 (0.885–3.943)	0.101		
BMI, kg/m^2^	0.963 (0.915–1.013)	0.140		
IPF diagnosis	3.479 (1.659–7.294)	0.001	2.544 (1.106–5.853)	0.028
CTD-ILD diagnosis	0.689 (0.330–1.442)	0.323		
Sarcoidosis diagnosis	0.235 (0.055–0.993)	0.049		
FVC, % predicted	0.981 (0.958–1.004)	0.099		
DL_CO_, % predicted	0.949 (0.922–0.977)	<0.0001	0.945 (0.914–0.977)	0.001
6MWD < 300 m	1.612 (0.733–3.545)	0.235		
6MWT final SpO_2_ < 88%	2.055 (0.867–4.871)	0.102		
mPAP, mmHg	1.006 (0.946–1.069)	0.850		
RAP, mmHg	1.045 (0.961–1.137)	0.306		
sPAP, mmHg	1.005 (0.976–1.034)	0.757		
dPAP, mmHg	1.015 (0.948–1.087)	0.662		
PCWP, mmHg	1.060 (1.009–1.114)	0.020		
PVR, Wood units	1.025 (0.913–1.151)	0.670		
CI, L/min/m^2^	0.621 (0.401–0.963)	0.033		
Antifibrotic therapy	1.482 (0.606–3.620)	0.388		
Immunomodulator therapy	0.409 (0.182–0.918)	0.030		
PH-specific therapy	0.375 (0.180–0.784)	0.009		
Oxygen supplementation	1.673 (0.681–4.108)	0.262		

HR: hazard ratio; and 95% CI: 95% confidence interval. BMI: body mass index; IPF; idiopathic pulmonary fibrosis; CTD: connective tissue disease; ILD: interstitial lung disease; FVC: forced vital capacity; DLco: diffusion capacity of the lung for carbon monoxide; 6MWD: six-minute walk distance; 6MWT: six-minute walk test; SpO_2_: oxygen saturation by pulse oximetry; mPAP: mean pulmonary artery pressure; RAP: right atrial pressure; sPAP: systolic pulmonary artery pressure; dPAP: diastolic pulmonary artery pressure; PCWP: pulmonary capillary wedge pressure; PVR: pulmonary vascular resistance; CI: cardiac index; PH: pulmonary hypertension.
